# Gastrodin Inhibits Expression of Inducible NO Synthase, Cyclooxygenase-2 and Proinflammatory Cytokines in Cultured LPS-Stimulated Microglia via MAPK Pathways

**DOI:** 10.1371/journal.pone.0021891

**Published:** 2011-07-12

**Authors:** Ji-Nan Dai, Yi Zong, Lian-Mei Zhong, Yue-Min Li, Wei Zhang, Li-Gong Bian, Qing-Long Ai, Yi-Dan Liu, Jun Sun, Di Lu

**Affiliations:** 1 Department of Anatomy, Kunming Medical University, Kunming, Yunnan, China; 2 Department of Neurology, the First Affiliated Hospital of Kunming Medical University, Kunming, Yunnan, China; 3 Kunming Pharmaceutical Corporation, Kunming, Yunnan, China; 4 Rehabilitation Engineering Research Laboratory, Biomedicine Engineering Research Centre, Kunming Medical University, Kunming, Yunnan, China; Universidad Federal de Santa Catarina, Brazil

## Abstract

**Background:**

Microglial activation plays an important role in neurodegenerative diseases by producing several proinflammatory enzymes and proinflammatory cytokines. The phenolic glucoside gastrodin, a main constituent of a Chinese herbal medicine, has been known to display anti-inflammatory properties. The current study investigates the potential mechanisms whereby gastrodin affects the expression of potentially pro-inflammatory proteins by cultured murine microglial BV-2 cells stimulated with lipopolysaccharide (LPS).

**Methodology/Principal Findings:**

BV-2 cells were pretreated with gastrodin (30, 40, and 60 µM) for 1 h and then stimulated with LPS (1 µg/ml) for another 4 h. The effects on proinflammatory enzymes, inducible nitric oxide synthase (iNOS) and cyclooxygenase-2 (COX-2), and proinflammatory cytokines, tumor necrosis factor-α (TNF-α), and interleukin-1β (IL-1β), are analysed by double-immunofluorescence labeling and RT-PCR assay. To reveal the mechanisms of action of gastrodin we investigated the involvement of mitogen-activated protein kinases (MAPKs) cascades and their downstream transcription factors, nuclear factor-κB (NF-κB) and cyclic AMP-responsive element (CRE)-binding protein (CREB). Gastrodin significantly reduced the LPS-induced protein and mRNA expression levels of iNOS, COX-2, TNF-α, IL-1β and NF-κB. LPS (1 µg/ml, 30 min)-induced phosphorylation of extracellular signal-regulated kinase 1/2 (ERK1/2), c-Jun N-terminal protein kinase (JNK) and p38 mitogen-activated protein kinase (p38 MAPK) and this was inhibited by pretreatment of BV-2 cells with different concentrations of gastrodin (30, 40, and 60 µM). In addition, gastrodin blocked LPS-induced phosphorylation of inhibitor κB-α (IκB-α) (and hence the activation of NF-κB) and of CREB, respectively.

**Conclusion and Implications:**

This study indicates that gastrodin significantly attenuate levels of neurotoxic proinflammatory mediators and proinflammatory cytokines by inhibition of the NF-κB signaling pathway and phosphorylation of MAPKs in LPS-stimulated microglial cells. Arising from the above, we suggest that gastrodin has a potential as an anti-inflammatory drug candidate in neurodegenerative diseases.

## Introduction

Microglial cells are the resident macrophage-like population of cells, which has been proposed to play a pivotal role in the innate immune response in the central nervous system (CNS) [1]. Although activated microglia scavenge dead cells from the CNS and secrete different neurotrophic factors for neuronal survival [2], [3], [4], it is believed that severe activation causes various autoimmune responses leading to neuronal death and brain injury [5], [6]. Activation of microglia has been implicated in the pathogenesis of variety of neurodegenerative diseases, including multiple sclerosis, Parkinson's disease, Huntington's disease, and Alzheimer's disease [7], [8], [9]. Activation of microglia and consequent release of proinflammatory and/or cytotoxic factors such as tumor necrosis factor-α (TNF-α), interleukin-1β (IL-1β), nitric oxide (NO), reactive oxygen species (ROS), inducible nitric oxide synthase (iNOS), and cyclooxygenase-2 (COX-2) are believed to contribute to neurodegenerative processes [10], [11], [12], [13].

iNOS is not normally expressed in the brain, but inflammatory stimuli such as lipopolysaccharide (LPS) and cytokines cause its expression in microglia and astrocytes [14]. Once expressed, iNOS produces high levels of NO continuously [15]. A number of studies have shown that the iNOS along with the release of NO by activated microglia contributes to progress neurodegeneration and aggravate neuronal diseases [16]. NO activates COX-2 resulting in the increased release of proinflammatory prostaglandins [17]. Induction of COX-2 expression and enzymatic activity can be associated with the management of inflammation and several neuronal diseases [18].

It has recently been suggested that the activation of microglia can increase neurotoxicity through the production of proinflammatory and cytotoxic factors in neuron-glia cultures treated with LPS, β-amyloid, glutamate, and arachidonate [19]. One of these widely used stimuli is LPS, a bacterial endotoxin used to study experimentally induced infection, inflammation, or tissue damage, as well as the biochemistry of inflammatory responses. LPS activates nuclear factor-κB (NF-κB), cyclic AMP responsive element-binding protein (CREB) and mitogen-activated protein kinases (MAPKs) family, which are classified into at least three components: extracellular signal-regulated kinases (ERKs), c-Jun N-terminal kinase (JNK), and p38 MAPK [20], which have been implicated in the release of immune-related cytotoxic factors such as iNOS, COX-2, and proinflammatory cytokines [12], [21].

**Figure 1:**
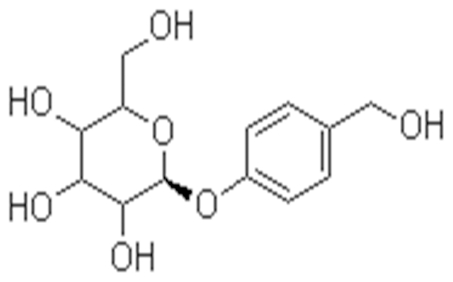
Structure of gastrodin.

The phenolic glucoside gastrodin (Figure 1), the main active ingredient of an ancient Chinese herb Tianma (Gastrodia elata Bl.), is considered to have several beneficial properties. Gastrodin has been suggested to be effective as an anticonvulsant, analgesic, and sedative effective against vertigo, general paralysis, epilepsy, and tetanus [22]. Gastrodin could penetrate through the blood-brain barrier into brain, and it was rapidly decomposed to *p*-hydroxybenzyl alcohol (HBA) in brain, liver, and blood [23]. It is well known that gastrodin ameliorates cerebral damage after transient focal cerebral ischemia by promoting the ability to reduce ROS damage *in vivo* and hippocampal neuronal death and excitotoxicity *in vitro* [24] and has a neuroprotective action against hypoxia in the cultured cortical neuron [25]. There are numerous reports in the literature show that gastrodin and HBA, an aglycone of gastrodin, may improve learning and facilitate memory consolidation and retrieval [26], [27]. There is, however, no report on the bioactive principles and the detailed mechanisms responsible for the anti-inflammatory activity of this herbal plant.

In the present study, we attempted to elucidate the anti-inflammatory potential of gastrodin by investigating the effect of gastrodin on the inflammatory response induced by LPS in murine microglial BV-2 cells. To further investigate the underlying mechanisms, the involvement of CREB, NF-κB and MAPKs was also examined. The present study provides information revealing gastrodin as a potential candidate compound with anti-inflammatory actions and suggests a scientific basis for further investigation of gastrodin against neuroinflammatory conditions.

## Results

### Gastrodin inhibits LPS-stimulated expression of iNOS and COX-2 proteins and mRNA in BV-2 cells

**Figure 2:**
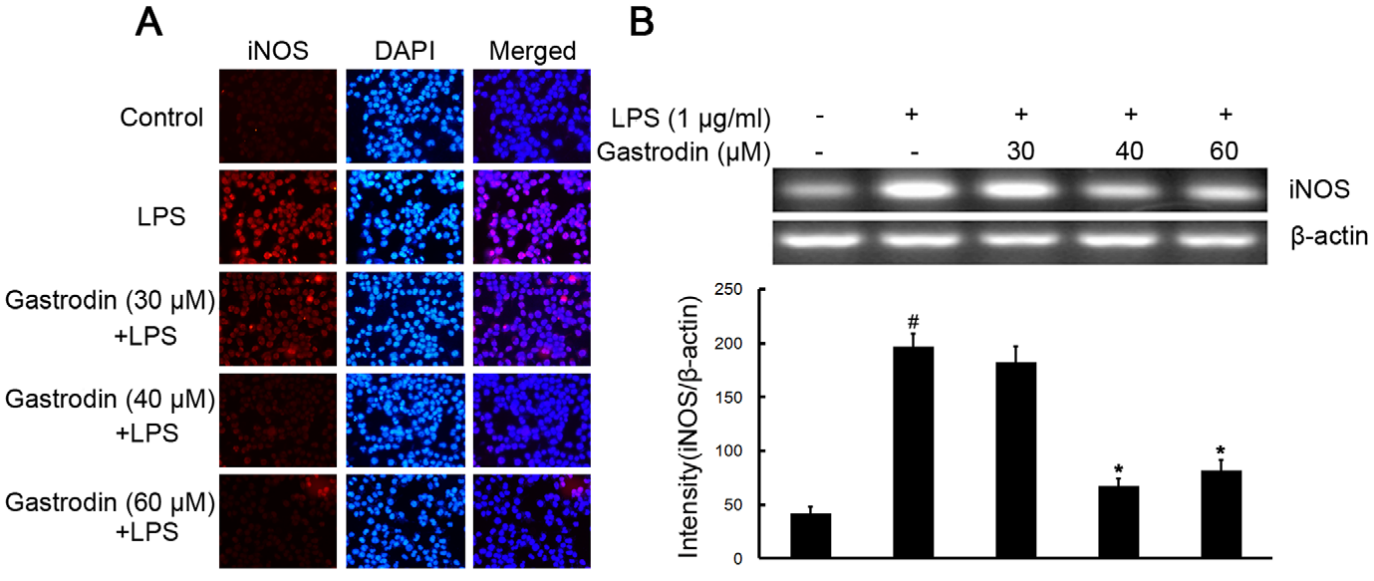
Inhibitory effects of gastrodin on the LPS-induced expression of protein and mRNA for iNOS in BV-2 cells. Approximately 1×10^6^ cells/ml were seeded in six-well plates and incubated until 80% confluency. Cells were pre-treated with gastrodin (30, 40, and 60 µM) for 1 h, then exposed to 1 µg/ml LPS for 4 h. The levels of protein and the corresponding mRNA were determined by double-immunofluorescence labeling and RT-PCR as described in the Methods. Panel A shows the immunofluorenscence images for protein expression of iNOS and Panel B shows the corresponding mRNA data. The relative mRNA level was quantified by scanning densitometry and normalized to β-actin mRNA. The values shown are mean ± SEM of data from three independent experiments. ^#^ Significant compared with control alone, *p*<0.05. ^*^Significant compared with LPS alone, *p*<0.05.

To investigate the effect of gastrodin on LPS-stimulated microglial activation, BV-2 cells were stimulated with LPS (1 µg/ml) which resulted in increase of the protein and mRNA levels of iNOS (Figure 2). Pre-treatment with gastrodin (30, 40, and 60 µM) notably inhibited dose-dependently iNOS protein and mRNA levels (Figure 2), compared with LPS-treated control.

**Figure 3:**
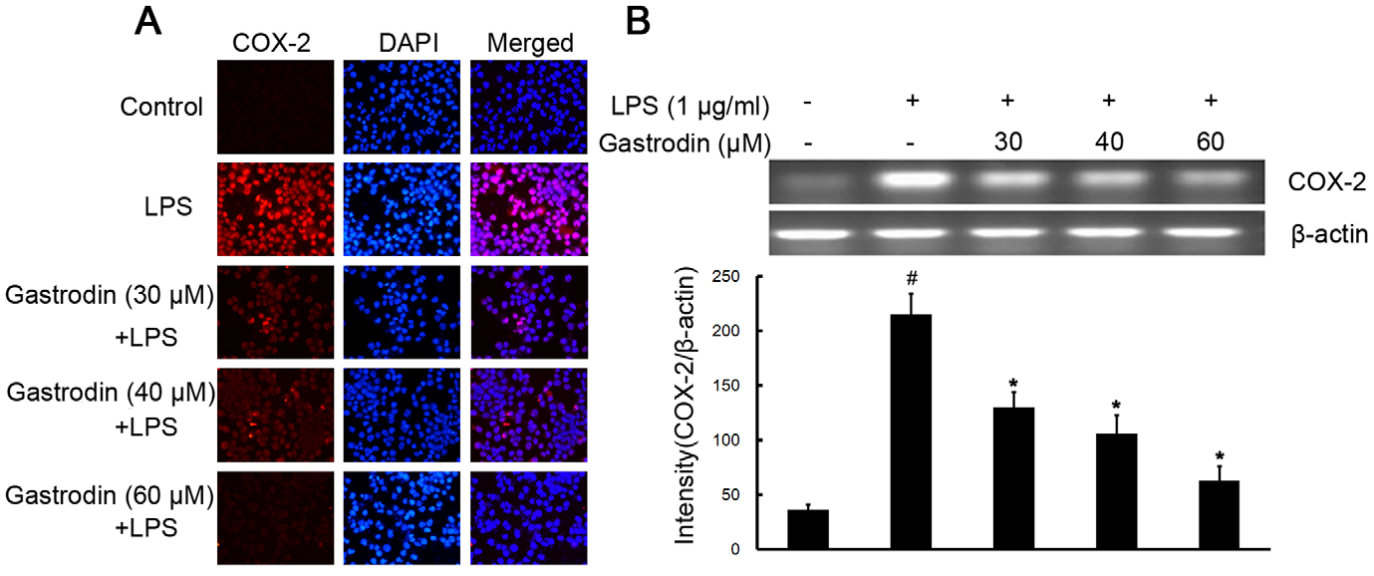
Inhibitory effects of gastrodin on the LPS-induced expression of protein and mRNA for COX-2 in BV-2 cells. Approximately 1×10^6^ cells/ml were seeded in six-well plates and incubated until 80% confluency. Cells were pre-treated with gastrodin (30, 40, and 60 µM) for 1 h, then exposed to 1 µg/ml LPS for 4 h. The levels of protein and the corresponding mRNA were determined by double-immunofluorescence labeling and RT-PCR as described in the Methods. Panel A shows the immunofluorenscence images for protein expression of COX-2 and Panel B shows the corresponding mRNA data. The relative mRNA level was quantified by scanning densitometry and normalized to β-actin mRNA. The values shown are mean ± SEM of data from three independent experiments. ^#^ Significant compared with control alone, *p*<0.05. ^*^Significant compared with LPS alone, *p*<0.05.

Effect of gastrodin on another proinflammatory enzyme, COX-2 was examined by double-immunofluorescence labeling and RT-PCR assay. Stimulation of BV-2 cells with LPS led to increased expression of protein and mRNA for COX-2 (Figure 3). Gastrodin dose-dependently decreased the increased expression of COX-2 protein and mRNA stimulated by LPS (Figure 3).

### Gastrodin attenuates LPS-stimulated the production of the proinflammatory cytokines TNF-α and IL-1β at the transcriptional and translational levels in BV-2 cells

**Figure 4:**
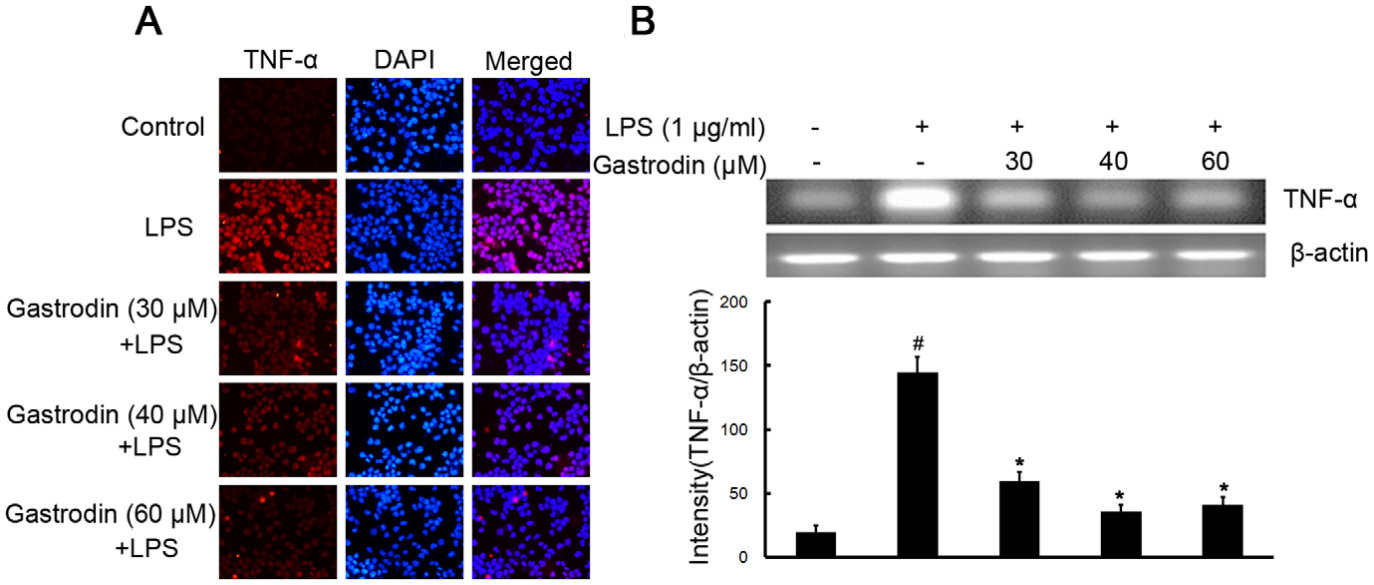
Inhibitory effects of gastrodin on the LPS-induced expression of protein and mRNA for TNF-α in BV-2 cells. Approximately 1×10^6^ cells/ml were seeded in six-well plates and incubated until 80% confluency. Cells were pre-treated with gastrodin (30, 40, and 60 µM) for 1 h, then exposed to 1 µg/ml LPS for 4 h. The levels of protein and the corresponding mRNA were determined by double-immunofluorescence labeling and RT-PCR as described in the Methods. Panel A shows the immunofluorenscence images for protein expression of TNF-α and Panel B shows the corresponding mRNA data. The relative mRNA level was quantified by scanning densitometry and normalized to β-actin mRNA. The values shown are mean ± SEM of data from three independent experiments. ^#^ Significant compared with control alone, *p*<0.05. ^*^Significant compared with LPS alone, *p*<0.05.

**Figure 5:**
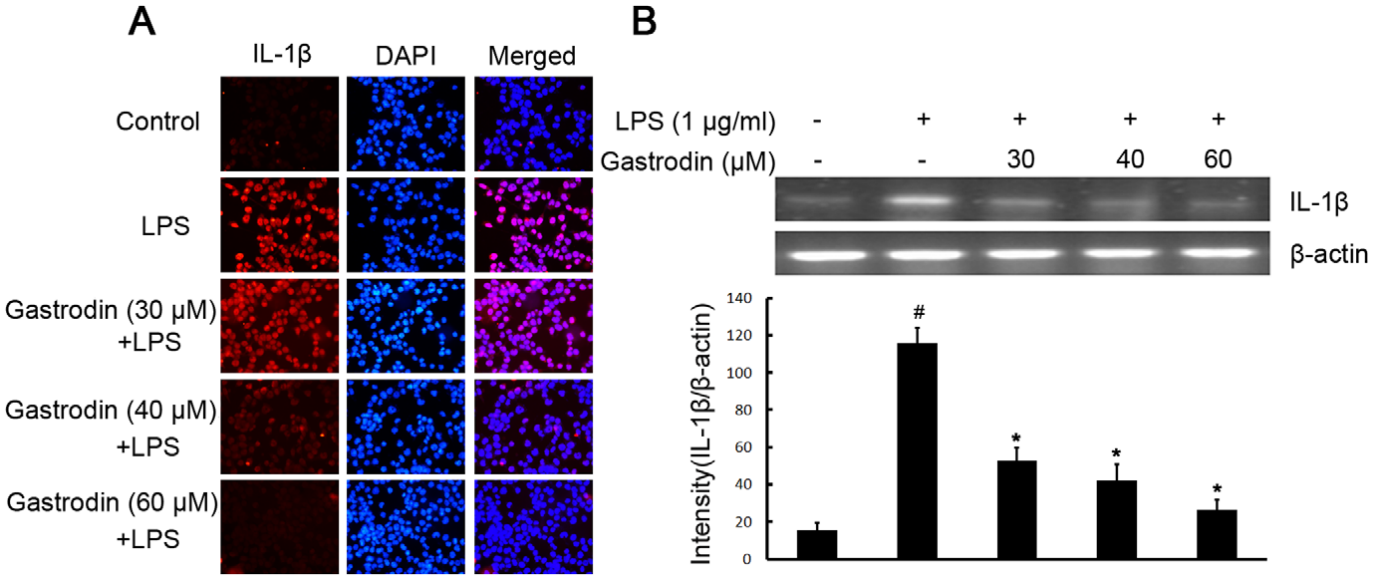
Inhibitory effects of gastrodin on the LPS-induced expression of protein and mRNA for IL-1β in BV-2 cells. Approximately 1×10^6^ cells/ml were seeded in six-well plates and incubated until 80% confluency. Cells were pre-treated with gastrodin (30, 40, and 60 µM) for 1 h, then exposed to 1 µg/ml LPS for 4 h. The levels of protein and the corresponding mRNA were determined by double-immunofluorescence labeling and RT-PCR as described in the Methods. Panel A shows the immunofluorenscence images for protein expression of IL-1β and Panel B shows the corresponding mRNA data. The relative mRNA level was quantified by scanning densitometry and normalized to β-actin mRNA. The values shown are mean ± SEM of data from three independent experiments. ^#^ Significant compared with control alone, *p*<0.05. ^*^Significant compared with LPS alone, *p*<0.05.

To investigate whether gastrodin represses the production of these proinflammatory cytokines, which play central roles in inflammatory disease, BV-2 cells were stimulated with LPS (1 µg/ml) in the presence or absence of gastrodin (30, 40, and 60 µM). After treatment with LPS, the protein levels of the cytokines in BV-2 cells were measured. The protein levels of TNF-α and IL-1β increased in LPS-stimulated BV-2 cells. Pre-treatment with gastrodin resulted in a significant decrease in cytokine production (Figure 4A and 5A).

To further investigate whether the inhibitory effect of gastrodin on TNF-α and IL-1β production is due to the reduced expression of cognate genes, the effect of gastrodin on mRNA expression of TNF-α and IL-1β was assessed in LPS-stimulated BV-2 cells. As shown in Figure 4B and 5B, the mRNA expression of these inflammatory mediators was very low or hardly detectable in unstimulated BV-2 cells. However, BV-2 cells expressed high levels of TNF-α and IL-1β mRNA when stimulated with LPS (1 µg/ml). Furthermore, gastrodin suppressed LPS-induced expression of these genes in a concentration-dependent manner. In contrast, the level of β-actin mRNA was not affected by LPS and gastrodin treatment (Figure 4B and 5B).

### Gastrodin suppresses LPS-induced expression of NF-κB/RelA protein, phosphorylation of IκB-α and CREB in BV-2 cells

**Figure 6:**
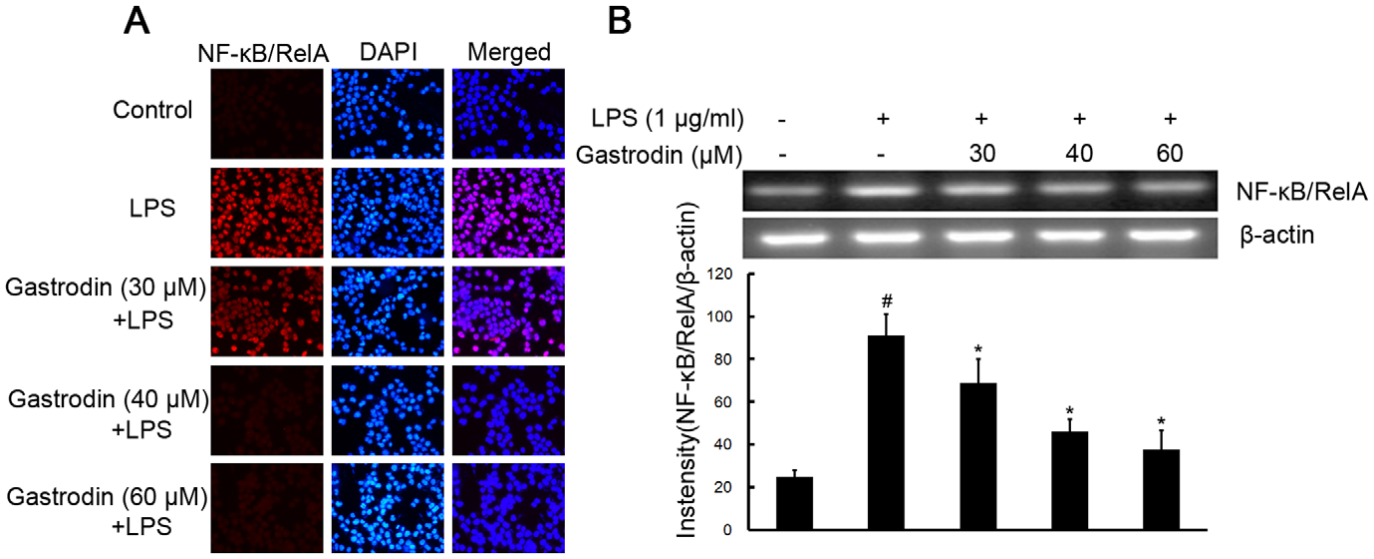
Inhibitory effects of gastrodin on the LPS-induced expression of protein and mRNA for NF-κB/RelA in BV-2 cells. Approximately 1×10^6^ cells/ml were seeded in six-well plates and incubated until 80% confluency. Cells were pre-treated with gastrodin (30, 40, and 60 µM) for 1 h, then exposed to 1 µg/ml LPS for 4 h. The levels of protein and the corresponding mRNA were determined by double-immunofluorescence labeling and RT-PCR as described in the Methods. Panel A shows the immunofluorenscence images for protein expression of NF-κB/RelA and Panel B shows the corresponding mRNA data. The relative mRNA level was quantified by scanning densitometry and normalized to β-actin mRNA. The values shown are mean ± SEM of data from three independent experiments. ^#^ Significant compared with control alone, *p*<0.05. ^*^Significant compared with LPS alone, *p*<0.05.

**Figure 7:**
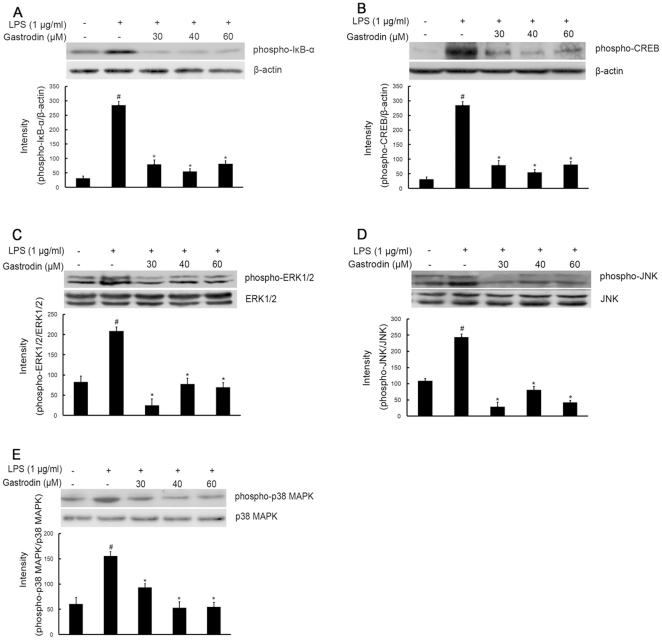
Inhibitory effects of gastrodin on the LPS-induced phosphorylation expression of IκB-α(A), CREB (B), ERK1/2 (C), JNK(D) and p38 MAPK (E) in BV-2 cells. Approximately 1×10^6^ cells/ml were seeded in six-well plates and incubated until 80% confluency. Cells were pre-treated with gastrodin (30, 40, and 60 µM) for 1 h, then exposed to 1 µg/ml LPS for 30 min. Cell lysates (50 µg protein) were prepared and subjected to Western blot analysis by using antibodies specific for phosphorylated forms of IκB-α, CREB, ERK1/2, JNK and p38 MAPK (shown as phospho-IκB-α, etc.) as described in the methods. Equivalent loading of cell lysates was determined by reprobing the blots with anti-β-actin, total ERK1/2, JNK or p38 MAPK antibodies. The relative protein levels were quantified by scanning densitometry and normalized to β-actin, total ERK1/2, JNK or p38 MAPK. The values shown are mean ± SEM of data from three independent experiments. ^#^ Significant compared with control alone, *p*<0.05. ^*^Significant compared with LPS alone, *p*<0.05.

To further elucidate the mechanisms of gastrodin on the inhibition of expression of iNOS, COX-2, and proinflammatory cytokines in microglia, the study examined the effect of gastrodin on NF-κB and CREB, two major transcription factors involved in the expression of these inflammatory mediators. The processes of NF-κB activation include IκB-α degradation through phosphorylation and a subsequent nuclear translocation of NF-κB. We determined whether the inhibitory effects of gastrodin occurred through the blockade of NF-κB activation in BV-2 cells. LPS induced expression of NF-κB/RelA protein and mRNA within 4 h after stimulation, which was inhibited by gastrodin (Figure 6). Meanwhile, as shown in Figure 7A, pre-treatment of gastrodin (30, 40, and 60 µM) suppressed the LPS induced phosphorylation of IκB-α.

CREB is the physiological substrate for MAPKs and stress-activated protein kinases-1 (MSK1), which is activated by ERK and p38 MAPK-mediated signaling in response to LPS. CREB activation was markedly stimulated by LPS, and this activation was significantly inhibited by pre-treatment with gastrodin, at all three concentrations (Figure 7B).

### Gastrodin decreases LPS-induced phosphorylation of MAPKs family in BV-2 cells

The effect of gastrodin on MAPKs, which are upstream signaling molecules in inflammatory reactions, was examined in the LPS-stimulated BV-2 cells. Western blot analysis was carried out using the phospho- or total forms of antibodies against the three MAPKs, ERK1/2, JNK, and p38 MAPK. It was observed that gastrodin (30, 40, and 60 µM) remarkably decreased the LPS-stimulated phosphorylation of ERK1/2 at 30 min, respectively, whereas it had no effect on the expression level of ERK1/2 in LPS-stimulated BV-2 cells. (Figure 7C). Meanwhile, gastrodin at all concentrations used significantly suppressed the phosphorylation of JNK and p38 MAPK, respectively, but did not affect the expression levels of JNK and p38 MAPK in LPS-stimulated BV-2 cells (Figure 7D and 7E).

## Discussion

Microglial activation has both beneficial and harmful effects on neuronal cell survival [28]. However, there are multiple lines of evidence suggesting that microglial activation is more associated with neurodegenerative diseases such as Alzheimer's disease and Parkinson's disease [29], [30]. Over-activation of microglia contributes to neurodegenerative processes through the production of various neurotoxic factors including free radicals and proinflammatory cytokines [31]. Therefore, inhibition of microglial activation may be a potential therapeutic strategy to reduce neuronal cell death. In fact, a number of anti-inflammatory agents, which inhibit microglial activation or production of proinflammatory mediators in some diseases in the central nervous system disease conditions, attenuate neuronal degeneration [32], [33]. The present study demonstrates, for the first time, that gastrodin, a constituent of the traditional Chinese herb Tianma (Gastrodin elata Bl.), significantly inhibits LPS-induced enhancement of expression of proinflammatory enzymes (iNOS and COX-2) and proinflammatory cytokines (TNF-α and IL-1β) in microglial cells, at both mRNA and protein levels, providing an underlying mechanism for the neuroprotective effects of gastrodin *in vitro*.

COX-1 is constitutively expressed in most tissues, while COX-2 is induced by an array of stimuli including cytokines, LPS, and growth factor in microglia and astrocytes [12], [34]. COX-2 is the key enzyme in the formation of prostaglandins (PGs) during inflammatory conditions [18]. Although the relationship between microglial activation and neurodegenerative disease is not completely understood, it is well accepted that microglial activation is involved in the pathogenesis of various neurological and neurodegenerative diseases [35], [36]. In addition, microglial cells in the healthy brain do not express iNOS, but they become activated to produce iNOS and release a large amount of NO following ischemic, traumatic, neurotoxic or inflammatory damage [12], [37], [38], [39]. Increased NO production in the brain by prolonged activation of microglial cells is associated with progression of neurodegenerative disease [14]. Therefore, any substance that can attenuate expression of iNOS and COX-2 would be beneficial for delaying the progression of neurodegenerative disease. In the present study, gastrodin (30, 40, and 60 µM) significantly inhibited the protein and mRNA expression of iNOS and COX-2 in a dose-dependent manner in LPS-stimulated BV-2 microglial cells, suggesting possible beneficial effects of gastrodin by attenuating the activation of microglial cells and subsequent production of inflammatory mediators.

TNF-α and IL-1β are two main proinflammatory cytokines that are produced by activated microglia during CNS inflammation. In the CNS, a number of stimuli, such as LPS, β-amyloid and traumatic brain injury have been shown to abundantly produce TNF-α and IL-1β [40], [41]. Overproduction of proinflammatory cytokines from activated microglial cells has a detrimental effect on neuronal cells. This study investigated whether gastrodin inhibits LPS-induced production of proinflammatory cytokines in BV-2 cells. Our data suggest that gastrodin inhibited LPS-induced production of TNF-α and IL-1β in a dose-dependent manner. Gastrodin also attenuated their mRNA expression in LPS-stimulated BV-2 cells. These results suggest that gastrodin inhibited the production of proinflammatory cytokines through the regulation of their gene transcriptional levels in activated BV-2 cells.

The transcriptional regulation of iNOS, COX-2 and inflammatory cytokines, such as TNF-α and IL-1β, is a tightly controlled event. A variety of transcription factors, including NF-κB and CREB, is known to be involved in the transcriptional regulation of these inflammatory mediators [42]. NF-κB and CREB are important regulators of the expression of these inflammatory mediators [43], [44], [45]. In un-stimulated cells, NF-κB is retained in the cytoplasm by binding to IκB-α. Activation of NF-κB occurs via phosphorylation of its endogenous inhibitor IκB-α that resulting in the release and nuclear translocation of active NF-κB. The molecular mechanisms underlying anti-inflammatory effect of gastrodin, which showed the most potent anti-inflammatory activity, were further studied. The results suggest that LPS induced expression of NF-κB/RelA protein within 4 h after stimulation, which was inhibited by gastrodin as determined by a double-immunofluorescence labeling and RT-PCR assay. Meanwhile, BV-2 cells were pre-treated with gastrodin for 1 h and then stimulated with LPS for 30 min. Gastrodin suppressed the LPS-induced phosphorylation of IκB-α and CREB. These results indicated that the inhibition of gastrodin on the expression of iNOS, COX-2 and proinflammatory cytokines is partially through the suppression of NF-κB/RelA expression, the phosphorylation of IκB-α and CREB in LPS-stimulated BV-2 cells.

MAPKs family has been shown to play important roles in LPS-induced iNOS, COX-2, and proinflammatory cytokines expression in many types of cells [46], [47], [48]. It also has been reported that LPS-induced expression of proinflammatory cytokines expression is mediated by MAPKs signal transduction pathway in BV-2 cells [34]. Therefore, we investigated the effect of gastrodin on activation (phosphorylation) of three MAPKs induced by LPS in BV-2 cells. In our study, LPS increased activation of MAPKs, including ERK1/2, JNK, and p38 MAPK, within 30 min after stimulation, whereas gastrodin decreased LPS-induced activation of MAPKs, which was accompanied by alterations in iNOS, COX-2, and proinflammatory cytokines. These results suggested that gastrodin-mediated attenuation of proinflammatory mediators is associated with down-regulation of the MAPK signaling pathway.

The present results have shown that gastrodin significantly suppressed LPS-upregulated expression levels of iNOS, COX-2, IL-1β and NF-κB/RelA, though not TNF-α, in BV-2 cells in a dose dependent manner; on the other hand, its inhibitory effects were not followed dose dependently in the mRNA expression of these biomarkers. The same phenomenon was manifested by the inhibitory effects on phosphorylation of IκB-α, CREB, MAPKs including ERK1/2, JNK and p38 MAPK. Hence, while the inhibitory effects of gastrodin on the above-mentioned various cytokines and enzymes linked to various signaling routes are unequivocal, the actual mechanistic link between them remains to be fully elucidated. In view of the fact that gastrodin did not inhibit phosphorylation of IκB-α or the various signaling routes in a dose dependent manner, the possibility that other pathways by which gastrodin can regulate the target proteins investigated should be considered. In addition, it needs to be pointed out that the inhibitory changes as observed in this study were based on a single time point. Considering the effects of LPS and gastrodin develop over a significant period of time, a time course study to investigate these time-related aspects would appear to be important.

In conclusion, we show here the inhibitory effects of gastrodin on LPS-induced proinflammatory mediators in microglial BV-2 cells. In this connection, gastrodin significantly attenuated the expression levels of neurotoxic proinflammatory mediators, including iNOS, COX-2, and proinflammatory cytokines (TNF-α and IL-1β) in LPS-stimulated microglial cells. This was accompanied by attenuation of expression levels of NF-κB/RelA protein and phosphorylation of IκB-α and CREB. Furthermore, levels of phosphorylated MAPKs, including ERK1/2, JNK, and p38 MAPK, were significantly decreased by pre-treatment with gastrodin in LPS-stimulated microglial cells. Taken together, these results indicate that gastrodin exerts its anti-inflammatory actions by inhibition of the NF-κB signaling pathway and phosphorylation of MAPKs. Arising from the above, we suggest that gastrodin is a potential an anti-inflammatory drug candidate in neurodegenerative diseases.

## Methods

### Cells and treatments

The immortalized mouse microglial cell line BV-2 was developed in the laboratory of Dr Blasi at the University of Perugia [49] and was a generous gift of Dr Cheng-gang Zou (School of Life Science, Yunnan University, Kunming, China). Cells were cultured in Dulbecco's modified Eagle's medium (DMEM; Gibco/BRL, Gaithersburg, MD, USA) containing 2% fetal bovine serum (Hyclone, Logan, UT, USA) and antibiotics (100 IU/ml penicillin and 100 µg/ml streptomycin; Sigma, St. Louis, MO, USA) at a density not exceeding 5×10^5^ cells/ml and maintained at 37°C in a humidified incubator with 5% CO_2_. To harvest BV-2 cells, cells were trypsinized (0.25% trypsin/EDTA in phosphate-buffered saline (PBS); Sigma, St. Louis, MO, USA), then centrifuged (400 g for 10 min) and resuspended in serum-free DMEM. Cells were counted with a hemocytometer and trypan blue staining (0.4% trypan blue in PBS; Sigma) showed more than 99% of the cells retained viability. Cells (approximately 1×10^6^ cells/ml) were seeded in six-well plates before being subjected to treatments. Five groups of BV-2 cells were subjected to various treatments. In group 1, the cells were incubated in serum-free DMEM. In group 2, the cells were treated with 1 µg/ml LPS (from *Escherichia coli*, Sigma). In groups 3, 4, and 5, the cells were treated with 30 µM, 40 µM, and 60 µM gastrodin (Kunming Pharmaceutical Corporation, Kunming, China) for 1 h and then stimulated with LPS (1 µg/ml). This time point was chosen to minimize the possibility of any direct interactions between gastrodin and LPS. Cell incubations were for 30 min–4 h, as indicated in the text.

### Double-immunofluorescence labeling assay

Various treated BV-2 cells were fixed with 4% paraformaldehyde in 0.1 M phosphate buffer (PB) for 15 min. After rinsing with PBS, the coverslips with adherent cells were used for double-immunofluorescence labeling assay. BV-2 cells were incubated with DAPI (dilution 1∶50,000; Sigma) plus goat anti-mouse iNOS (dilution 1∶500; Santa Cruz Biotechnology, Santa Cruz, CA, USA), goat anti-mouse COX-2 (dilution 1∶500; Santa Cruz Biotechnology), goat anti-rabbit TNF-α polyclonal antibody (dilution 1∶500; Chemicon, Temecula, CA, USA), goat anti-rabbit IL-1β (dilution 1∶500; Chemicon), or goat anti-rabbit NF-κB/RelA (dilution 1∶500; Santa Cruz Biotechnology). Subsequently, the cells were incubated with TRITC-conjugated secondary antibodies (Santa Cruz Biotechnology) for 1 h at room temperature. For negative controls, a set of culture slides was incubated under similar conditions without the primary antibodies. All images were captured with a fluorescence microscope (D80i; Nikon, Tokyo, Japan). The results are representative of three independent experiments.

### Reverse transcription-polymerase chain reaction (RT-PCR) analysis

Total RNA was prepared from BV-2 cells by using the Trizol® reagent (Invitrogen Corporation, Carlsbad, CA, USA) according to the manufacturer's protocol. Total RNA was reverse-transcribed by using the Superscript™-III kit (Invitrogen) with 2.5 µg total RNA and oligo dT. Primer sequences were as follows: iNOS, sense: 5′- CTGCAGCACTTGGATCAGGAACC TG -3′, antisense: 5′- GGGAGTAGCCTGTGTGCACCTGGAA -3′; COX-2, sense: 5′-TTGAAGACCAGGAGTACAGC-3′, antisense: 5′-GGTACAGTTCCATGACATCG-3′; TNF-α, sense: 5′-CGTCAGCCGATTTGCTATCT-3′, antisense: 5′-CGGACTCCGCAAA GTCTAAG-3′; IL-1β, sense: 5′-GCCCATCCTCTGTGACTCAT-3′, antisense: 5′-AGGCCAC AGGTATTTTGTCG-3′; NF-κB/RelA, sense: 5′-GCGTACACATTCTGGGGAGT-3′, antisense: 5′-CCGAAGCAGGAGCTATCAAC-3′; β-actin, sense: 5′-AGCCATGTACGTAGCCATCC-3′, antisense: 5′-GCTGTGGTGGTGAAGCTGTA-3′. PCR amplification of the resulting cDNA template was conducted by using the following conditions for 45 (TNF-α, IL-1β, NF-κB/RelA and β-actin), 36 (COX-2) or 27 (iNOS) cycles. After an initial denaturation step at 95°C for 15 min, temperature cycling was initiated. Each cycle consisted of denaturation at 94°C for 15 sec, annealing at 60°C for 25 sec, and elongation at 72°C for 20 sec (TNF-α, IL-1β, NF-κB/RelA and β-actin). After an initial denaturation step at 95°C for 5 min, temperature cycling was initiated. Each cycle consisted of denaturation at 94°C for 30 sec, annealing at 57°C for 45 sec, and elongation at 72°C for 30 sec (COX-2). After an initial denaturation step at 95°C for 5 min, temperature cycling was initiated. Each cycle consisted of denaturation at 94°C for 45 sec, annealing at 60°C for 45 sec, and elongation at 70°C for 1 min (iNOS). PCR products were analyzed on 1% agarose gels and stained with 1 mg/ml ethidium bromide. Images were captured with a Gel Doc 2000 image analyzer (Bio-Rad, Richmond, CA, USA). The results are representative of three independent experiments.

### Western blot analysis

BV-2 cells were plated overnight in 6 well plates at a density of 5×10^5^ cells per plate, then the cells were further incubated in the medium without 10% FBS for at least 4 h before treatments. Cells were harvested with ice-cold PBS and centrifuged at 16 000×*g* for 5 min at 4°C. Stimulated cells were lysed in ice-cold lysis buffer [62.5 mM Tris–HCl, pH 6.8, 25% glycerol, 2% sodium dodecyl sulphate (SDS), 0.01% bromphenol blue and 5% β-mercaptoethanol]. Cell lysates were centrifuged at 16 000×*g* for 5 min at 4°C, then the supernatants were collected. Protein content was determined by using the BCA protein assay (Pierce, Rockford, IL, USA). Equal amounts of protein (50 µg) were loaded per lane onto 10% SDS-polyacrylamide gel electrophoresis (SDS-PAGE) and transferred onto immunoblot polyvinylidene difluoride membranes (Chemicon). The membranes were blocked with 5% non-fat milk in Tris-buffered saline containing 0.1% Tween 20 (TBS-T) for 2 h at room temperature and incubated separately with goat anti-rabbit antibodies for ERK1/2 and phospho-ERK1/2, JNK and phospho-JNK, p38 MAPK and phospho-p38 MAPK, phospho-IκB-α, phospho-CREB and β-actin antibodies (1∶1000 dilution; Cell Signaling Technology, Danvers, MA, USA) that recognize different molecules under study for overnight at 4°C. The membranes were then washed three times for 15 min with TBS-T, and incubated with a 1∶2000 dilution of horseradish peroxidase-coupled secondary antibodies (Santa Cruz Biotechnology) for 2 h at room temperature. Blots were again washed three times for 5 min each in TBS-T and developed by the ECL® detection system (Santa Cruz Biotechnology). Membranes were exposed to Fuji Medical X-Ray Film (Fuji Photo Film Co., Ltd, Karagawa, Japan). The results are representative of three independent experiments.

### Statistical analysis

Statistical analysis of the data was carried out by one way analysis of variance (ANOVA) followed by Scheffe's post hoc test, using SPSS (SPSS Inc., Chicago, IL, USA). Summary data are shown as mean ± SEM (standard error of mean) obtained from three independent experiments. Values of *p*<0.05 were considered significant.

## References

[1] Olson JK, Miller SD (2004). Microglia initiate central nervous system innate and adaptive immune responses through multiple TLRs.. J Immunol.

[2] Nakajima K, Kohsaka S (1998). Functional roles of microglia in the central nervous system.. Hum Cell.

[3] Suzumura A, Takeuchi H, Zhang G, Kuno R, Mizuno T (2006). Roles of glia-derived cytokines on neuronal degeneration and regeneration.. Ann N Y Acad Sci.

[4] Roy A, Fung YK, Liu X, Pahan K (2006). Up-regulation of Microglial CD11b Expression by Nitric Oxide.. J Biol Chem.

[5] Ankeny DP, Popovich PG (2009). Mechanisms and implications of adaptive immune responses after traumatic spinal cord injury.. Neuroscience.

[6] Fontana A, Gast H, Reith W, Recher M, Birchler T (2010). Narcolepsy: autoimmunity, effector T cell activation due to infection, or T cell independent, major histocompatibility complex class II induced neuronal loss?. Brain.

[7] Tai YF, Pavese N, Gerhard A, Tabrizi SJ, Barker RA (2007). Imaging microglial activation in Huntington's disease.. Brain Res Bull.

[8] Weiner HL (2009). The challenge of multiple sclerosis: how do we cure a chronic heterogeneous disease?. Ann Neurol.

[9] Amor S, Puentes F, Baker D, van der Valk P (2010). Inflammation in neurodegenerative diseases.. Immunology.

[10] Nam KN, Koketsu M, Lee EH (2008). 5-Chloroacetyl-2-amino-1,3-selenazoles attenuate microglial inflammatory responses through NF-kappaB inhibition.. Eur J Pharmacol.

[11] Wang MJ, Huang HY, Chen WF, Chang HF, Kuo JS (2010). Glycogen synthase kinase-3β inactivation inhibits tumor necrosis factor-α production in microglia by modulating nuclear factor κB and MLK3/JNK signaling cascades.. J Neuroinflammation.

[12] Choi Y, Lee MK, Lim SY, Sung SH, Kim YC (2009). Inhibition of inducible NO synthase, cyclooxygenase-2 and interleukin-1beta by torilin is mediated by mitogen-activated protein kinases in microglial BV2 cells.. Br J Phamrmacol.

[13] Cao Q, Li P, Lu J, Dheen ST, Kaur C (2010). Nuclear factor-kappaB/p65 responds to changes in the Notch signaling pathway in murine BV-2 cells and in amoeboid microglia in postnatal rats treated with the gamma-secretase complex blocker DAPT.. J Neurosci Res.

[14] Murphy S (2000). Production of nitric oxide by glial cells: regulation and potential roles in the CNS.. Glia.

[15] Bal-Price A, Matthias A, Brown GC (2002). Stimulation of the NADPH oxidase in activated rat microglia removes nitric oxide but induces peroxynitrite production.. J Neurochem.

[16] Wang JY, Shum AY, Ho YJ, Wang JY (2003). Oxidative neurotoxicity in rat cerebral cortex neurons: synergistic effects of H_2_O_2_ and NO on apoptosis involving activation of p38 mitogen-activated protein kinase and caspase-3.. J Neurosci Res.

[17] Salvemini D, Misko TP, Masferrer JL, Seibert K, Currie MG (1993). Nitric oxide activates cyclooxygenase enzymes.. Proc Natl Acad Sci USA.

[18] Liang X, Wu L, Wang Q, Hand T, Bilak M (2007). Function of COX-2 and prostaglandins in neurological disease.. J Mol Neurosci.

[19] Brown GC, Neher JJ (2010). Inflammatory neurodegeneration and mechanisms of microglial killing of neurons.. Mol Neurobiol.

[20] Martindale JL, Holbrook NJ (2002). Cellular response to oxidative stress: signaling for suicide and survival.. J Cell Physiol.

[21] Ajmone-Cat MA, De Simone R, Nicolini A, Minghetti L (2003). Effects of phosphatidylserine on p38 mitogen activated protein kinase, cyclic AMP responding element binding protein and nuclear factor-kappaB activation in resting and activated microglial cells.. J Neurochem.

[22] Ojemann LM, Nelson WL, Shin DS, Rowe AO, Buchanan RA (2006). Tian ma, an ancient Chinese herb, offers new options for the treatment of epilepsy and other conditions.. Epilepsy Behav.

[23] Lin LC, Chen YF, Lee WC, Wu YT, Tsai TH (2008). Pharmacokinetics of gastrodin and its metabolite p-hydroxybenzyl alcohol in rat blood, brain and bile by microdialysis coupled to LC-MS/MS.. J Pharm Biomed Anal.

[24] Zeng X, Zhang S, Zhang L, Zhang K, Zheng X (2006). A study of the neuroprotective effect of the phenolic glucoside gastrodin during cerebral ischemia in vivo and in vitro.. Planta Med.

[25] Xu X, Lu Y, Bie X (2007). Protective effects of gastrodin on hypoxia-induced toxicity in primary cultures of rat cortical neurons.. Planta Med.

[26] Hsieh MT, Wu CR, Chen CF (1997). Gastrodin and p-hydroxybenzyl alcohol facilitate memory consolidation and retrieval, but not acquisition, on the passive avoidance task in rats.. J Ethnopharmacol.

[27] Wu CR, Hsieh MT, Huang SC, Peng WH, Chang YS (1996). Effects of Gastrodia elata and its active constituents on scopolamine-induced amnesia in rats.. Planta Med.

[28] Nakajima K, Kohsaka S (2001). Microglia: activation and their significance in the central nervous system.. J Biochem.

[29] Long-Smith CM, Sullivan AM, Nolan YM (2009). The influence of microglia on the pathogenesis of Parkinson's disease.. Prog Neurobiol.

[30] Lue LF, Kuo YM, Beach T, Walker DG (2010). Microglia activation and anti-inflammatory regulation in Alzheimer's disease.. Mol Neurobiol.

[31] Klegeris A, McGeer EG, McGeer PL (2007). Therapeutic approaches to inflammation in neurodegenerative disease.. Curr Opin Neurol.

[32] Asanuma M, Miyazaki I, Ogawa N (2004). Neuroprotective effects of nonsteroidal anti-inflammatory drugs on neurodegenerative diseases.. Curr Pharm Des.

[33] Tansey MG, Goldberg MS (2010). Neuroinflammation in Parkinson's disease: its role in neuronal death and implications for therapeutic intervention.. Neurobiol Dis.

[34] Lim JY, Won TJ, Hwang BY, Kim HR, Hwang KW (2010). The new diterpene isodojaponin D inhibited LPS-induced microglial activation through NF-kappaB and MAPK signaling pathways.. Eur J Pharmacol.

[35] Giovannini MG, Scali C, Prosperi C, Bellucci A, Pepeu G (2003). Experimental brain inflammation and neurodegeneration as model of Alzheimer's disease: protective effects of selective COX-2 inhibitors.. Int J Immunopathol Pharmacol.

[36] Miller RL, James-Kracke M, Sun GY, AY (2009). Oxidative and inflammatory pathways in Parkinson's disease.. Neurochem Res.

[37] Hanisch UK (2002). Microglia as a source and target of cytokines.. Glia.

[38] Son HY, Han HS, Jung HW, Park YK (2009). Panax notoginseng attenuates the infarct volume in rat ischemic brain and the inflammatory response of microglia.. J Pharmacol Sci.

[39] Lee MJ, Yang CH, Jeon JP, Hwang M (2009). Protective effects of isoliquiritigenin against methamphetamine-induced neurotoxicity in mice.. J Pharmacol Sci.

[40] Nam KN, Park YM, Jung HJ, Lee JY, Min BD (2010). Anti-inflammatory effects of crocin and crocetin in rat brain microglial cells.. Eur J Pharmacol.

[41] Szczepanik AM, Ringheim GE (2003). IL-10 and glucocorticoids inhibit Abeta(1–42)- and lipopolysaccharide-induced pro-inflammatory cytokine and chemokine induction in the central nervous system.. J Alzheimers Dis.

[42] Vaillancourt F, Morquette B, Shi Q, Fahmi H, Lavigne P (2007). Differential regulation of cyclooxygenase-2 and inducible nitric oxide synthase by 4-hydroxynonenal in human osteoarthritic chondrocytes through ATF-2/CREB-1 transactivation and concomitant inhibition of NF-kappaB signaling cascade.. J Cell Biochem.

[43] Yabe T, Sanagi T, Schwartz JP, Yamada H (2005). Pigment epithelium-derived factor induces pro-inflammatory genes in neonatal astrocytes through activation of NF-kappa B and CREB.. Glia.

[44] Vitor CE, Figueiredo CP, Hara DB, Bento AF, Mazzuco TL (2009). Therapeutic action and underlying mechanisms of a combination of two pentacyclic triterpenes, alpha- and beta-amyrin, in a mouse model of colitis.. Br J Pharmacol.

[45] Spooren A, Kooijman R, Lintermans B, Van Craenenbroeck K, Vermeulen L (2010). Cooperation of NF-kappaB and CREB to induce synergistic IL-6 expression in astrocytes.. Cell Signal.

[46] Matsuda T, Omori K, Vuong T, Pascual M, Valiente L (2005). Inhibition of p38 pathway suppresses human islet production of pro-inflammatory cytokines and improves islet graft function.. Am J Transplant.

[47] Hatziieremia S, Gray AI, Ferro VA, Paul A, Plevin R (2006). The effects of cardamonin on lipopolysaccharide-induced inflammatory protein production and MAP kinase and NF-kappaB signaling pathways in monocytes/macrophages.. Br J Pharmacol.

[48] Chen SR, Xu XZ, Wang YH, Chen JW, Xu SW (2010). Icariin derivative inhibits inflammation through suppression of p38 mitogen-activated protein kinase and nuclear factor-kappaB pathways.. Biol Pharm Bull.

[49] Blasi E, Barluzzi R, Bocchini V, Mazzolla R, Bistoni F (1990). Immortalization of murine microglial cells by a v-raf/v-myc carrying retrovirus.. J Neuroimmunol.

